# Differences in Efficacy between Antibacterial Lock Therapy and the Standard of Care for CVC-Related Infections: A Systematic Review and Meta-Analysis

**DOI:** 10.3390/clinpract14040124

**Published:** 2024-08-09

**Authors:** Vincenzo Calabrese, Alessandra Farina, Veronica Maressa, Valeria Cernaro, Guido Gembillo, Roberta Maria Messina, Elisa Longhitano, Cinzia Ferio, Emanuele Venanzi Rullo, Domenico Santoro

**Affiliations:** 1 Unit of Nephrology and Dialysis, Department of Clinical and Experimental Medicine, University Hospital “G. Martino”, University of Messina, 98124 Messina, Italy; veronicamaressa@virgilio.it (V.M.); vcernaro@unime.it (V.C.); guidogembillo@live.it (G.G.); robi.messina.rm@gmail.com (R.M.M.); elisa.longhitano@libero.it (E.L.); cinziaferio@gmail.com (C.F.); dsantoro@unime.it (D.S.); 2 Department of Clinical and Experimental Medicine, CDS Medicina e Chirurgia, University of Messina, 98124 Messina, Italy; alessandrafarina999@gmail.com; 3 Unit of Infectious Diseases, Department of Clinical and Experimental Medicine, University Hospital “G. Martino”, University of Messina, 98124 Messina, Italy; evenanzirullo@unime.it

**Keywords:** antibacterial lock therapy, CVC-related infections, meta-analysis, systematic review

## Abstract

**Background**: Central Venous Catheter (CVC)-related infections cannot always be solved by replacement, due to some vascular anomalies or an emergency status. This comprehensive, evidence-based review aimed to define the efficacy of antibacterial lock therapy (ALT) compared to the standard of care (SoC) in CVC-related infections. **Methods**: We performed a systematic search in PubMed, Embase, and Google Scholar, looking for randomized controlled trials (RCTs) and cohort or case-control observational studies. The eligible studies considered the subjects with a diagnosis of CVC-related infections treated with antibacterial lock therapy (ALT) compared to the standard of care (SoC). **Results:** Among 609 records at the end of the selection process, five articles, referring to observational studies, were included in this systematic review. In pooled analyses, including a total of 276 individuals, microbiological healing (OR 3.78; 95% CI; 2.03–7.03) showed significant differences between ALT and the SoC, with a follow-up varying from 2 weeks to 3 months. **Conclusions:** Our results suggested that ALT could improve the preservation of CVCs and could be considered when their replacement is not possible as a result of vascular problems. However, only observational studies were included and RCTs are needed to confirm these findings and to increase the level of evidence.

## 1. Introduction

Central Venous Catheters (CVCs) are medical devices made of biocompatible material, used for long-term vascular access in patients requiring complex intravenous therapies or continuous hemodynamic monitoring. These devices provide direct access to the large central veins, such as the superior vena cava or the right atrium of the heart, and allow drug administration, fluid administration, and blood sampling with lower risks of complications than peripheral venous routes.

CVCs have transformed the treatment of patients requiring prolonged venous access, becoming indispensable devices for the management of both acute and chronic patients, so much so that almost 18% of patients hospitalized with at least one intravascular catheter have a CVC [1].

One of the most common complications is related to central line-associated bloodstream infections (CLABSI) [2]. These are associated with various microorganisms, and treatment is based on their features and sensibility.

Antibiotic lock therapy (ALT) is a local treatment that has the aim of preserving the central venous catheter (CVC) when it is possible, as the implantation and removal of catheters are complex and expensive procedures. Furthermore, the available vascular sites are limited, especially in the pediatric population.

The standard of care of the management of CVCs includes intraluminal anticoagulant administration, whereas antibiotic lock therapy consists of the adding of local antibiotics in the highest concentrations to penetrate the biofilm. The antibiotics are chosen according to the microbiological species and sensibility.

Some conditions oblige CVC replacement in the presence of a CLABSI [2], such as severe sepsis, infective endocarditis, septic thrombophlebitis, or bacteremia persisting for more than 72 h despite adequate antimicrobial therapy, as well as a CLABSI due to S aureus; MDR Gram-negative bacilli, fungi, or mycobacteria; *Micrococcus* spp.; or *Propionibacterium*, once blood culture contamination is excluded by the repeated recovery of the pathogen from blood cultures [3].

Since catheter removal is not always possible in patients who require chronic catheterization or who have multiple catheters, lock therapy can be used with the goal of sparing catheter removal and sterilizing the lumen [4].

Although some RCTs and several observational studies have evaluated the prevention of CVC-related infections or microbiological healing, albeit without a comparison with the standard of care, we aimed to evaluate how effective ALT is, adding the few results in the literature to this systematic review.

This comprehensive, evidence-based review aimed to define the efficacy of antibacterial lock therapy (ALT) compared to the standard of care (SoC) for CVC-related infections.

## 2. Methods

### 2.1. Data Source and Search Strategy

This meta-analysis followed the Preferred Reporting Items for Systematic Reviews and Meta-Analyses (PRISMA) guidelines [5] and was conducted according to a pre-published protocol (CRD42021267985) [6]. The literature search was designed and performed by two authors independently (V.C. and A.F.). A third author entered the screening process for the studies where agreement was not reached. Automatic tools were used to detect duplicate articles. We performed a systematic, highly sensitive search in PubMed and Embase for English language articles without any time restrictions up to 5 October 2023 (Supplementary Table S1). The gray literature was screened through Google Scholar, SCOPUS, and clinicaltrials.gov. The PICO model was defined as follows: a CVC-related infection (population), treated with antimicrobial lock therapy (interventions) vs. the standard of care (comparison), resulting in microbiological healing and adverse events (outcome). The studies evaluating the prevention of CVC-related infections or without comparison with the standard of care were excluded.

### 2.2. Study Selection and Data Extraction

We included randomized controlled trials (RCTs) and observational studies testing the effects of antibacterial therapy, including various antibacterial families, in patients with a diagnosis of CVC-related infections, without sex restrictions. Studies were included if they provided information on the outcomes of interest, such as (1) microbiological healing, (2) adverse events.

Studies were excluded if a comparison was performed for the prevention of infections, which was considered to be an exclusion criterion. Studies were also excluded if alternative care, homeopathic medicine, and phytotherapies were administered.

The articles were screened by the titles and abstracts by two independent investigators (V.C. and A.F.), excluding the studies not pertinent to the topic, and the full texts were subsequently assessed to determine their eligibility according to the pre-specified inclusion/exclusion criteria. Any disagreement on the study judgments was discussed with a third author (V.M.), who was not involved in the selection process.

Reviews, letters, case reports, and abstracts were excluded from the analyses but were screened for potential additional references.

### 2.3. Data Analysis

Pooled meta-analyses were carried out on the outcomes, when the data were provided in a suitable format and by more than two studies. Where a meta-analysis was not applicable, a qualitative synthesis was performed. The Odds Ratio (OR), computing a 95% confidence interval (CI), was calculated for the dichotomous outcomes. The data were pooled using the fixed effects model and analyzed with the random effects method to guarantee the strength of the model. We tested for heterogeneity using the χ^2^ statistic related to freedom degrees, with an alpha of 0.05 considered to represent statistical significance. In addition, the Cochrane-I^2^ was used to assess the degree of heterogeneity. I^2^ values of 25%, 50%, and 75% were assumed to correspond to low, medium, and high levels of heterogeneity, respectively. The study characteristics are reported in Table 1, and the results are summed up in the forest plot. To explore a possible source of heterogeneity, we performed sensitivity analyses according to the participants’ characteristics, different interventions, and the study durations. GRADE was not computed due to observational studies not being included and the quality of evidence being very low (GRADEpro GDT 2015) [7].

The risk of bias was not computed, because only observational studies were included by the end of the screening process.

The statistical analyses were performed using the Review Manager (RevMan; Version 5.4.) software.

Where a meta-analysis was not possible, due to the heterogeneity of the data report on the various included studies, a qualitative description was performed.

### 2.4. Quality and Risk of Bias Assessment

The quality of RCTs was assessed by using the checklist developed by the Cochrane Renal Group, which evaluates the presence of potential selection bias (random sequence generation and allocation concealment), performance bias (blinding of investigators and participants), detection bias (blinding of outcome assessors), attrition bias (incomplete outcome data), reporting bias (selective reporting), and possible other sources of bias. For observational studies, the complete quantitative risk of bias was not computed. However, selection bias, reporting bias, and incomplete outcome data will be described.

## 3. Results

### 3.1. Search Results

**Figure 1:**
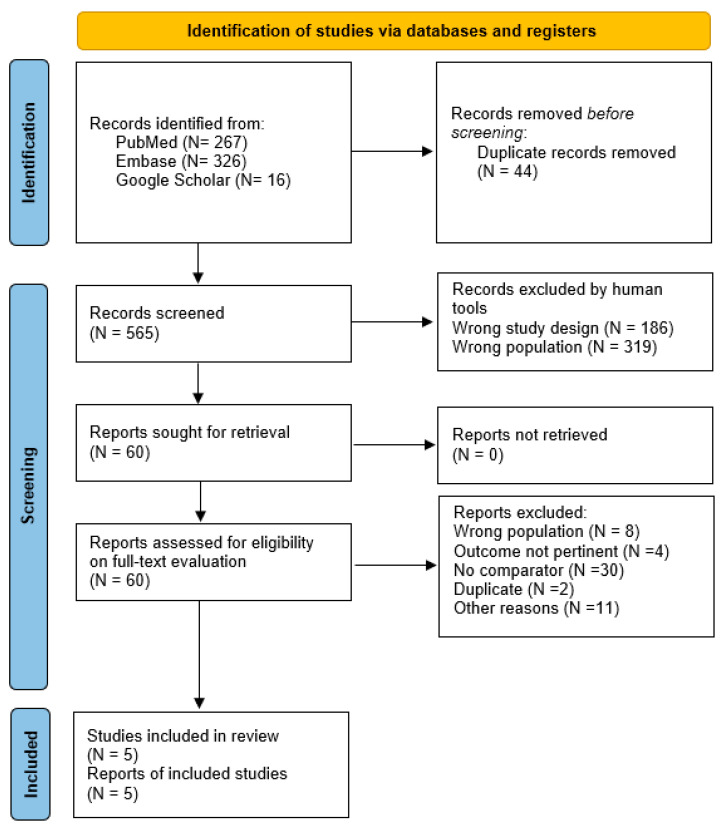
Study selection flowchart.

A flowchart summarizes the selection process (Figure 1). Six hundred and six references were retrieved. By screening the titles and abstracts, a total of 60 citations were selected for full-text evaluation. Among these, five articles [8,9,10,11,12] were reviewed in detail and included in this review. The other 55 articles were excluded for the following reasons: (1) dealing with other populations or not reporting outcomes pertinent to the topic (N = 12), (2) wrong intervention or no comparator (N = 30), (3) duplicates (N = 2), (4) various reasons (wrong publication type, only abstract) (N = 11).

### 3.2. Study Characteristics

All the observational studies had a parallel design. All the studies were published after 2003. The final population analyzed in this review included 276 patients, but the sample size of the studies was variable, spanning from 39 (Dannemberg C 2003 [10]) to 86 (Andres Blanco 2022 [8]) CVC-related infections. All the studies enrolled both male and female patients. Among these studies, the male gender spanned from 33% (Blanco-Di Matteo [9]) to 57% (Dannemberg C 2003 [10]). The mean age of the patients ranged from ~11 to ~70 years. The follow-ups spanned from 14 to 180 days. Teicoplanin, Vancomycin, Gentamycin, Daptomycin, Ceftazidime, Hetanol, and Clindamicin were used as the antimicrobial agents and were compared to the standard of care (Heparin). Teicoplanin was administered in a concentration of 10 ng/mL; Daptomycin, 5 ng/mL; Vancomycin, 2–10 ng/mL; Gentamycin, 2 ng/mL; Ceftriaxone, 500 mg/L (corresponding to 500 ng/mL); and Ciprofloxacin, 2 ng/mL. Only Dannemberg [10] did not report the concentration for the ALT or the concentration of Heparin. In all the included studies, the antimicrobial agent was chosen based on the species and sensibility of the bacteria, and they differed for each study.

The patients were afferent by various wards. Indeed, Andres Blanco 2022 [8] and Blanco Di Matteo 2022 [9] included hemodialyzed patients, Fortun J 2006 [11] and Rijnders B.J. 2004 [12] included hospitalized patients with afferents by various wards, and Dannemberg 2003 [10] included pediatric oncological patients.

The main characteristics of the five included observational studies are summarized in Table 1.

### 3.3. Study Quality and Risk of Bias

We would have computed the risk of bias for RCTs, but only observational studies were included after the selection process. Thus, the risk of bias was not evaluated, because the random sequence generation, allocation concealment, blinding of participants, and blinding of outcome assessors were not evaluable. However, incomplete outcome data and selective reporting were not detected in the included articles. No missing data related to microbiological healing were retrieved.

#### 3.3.1. Microbiological Healing

**Figure 2:**
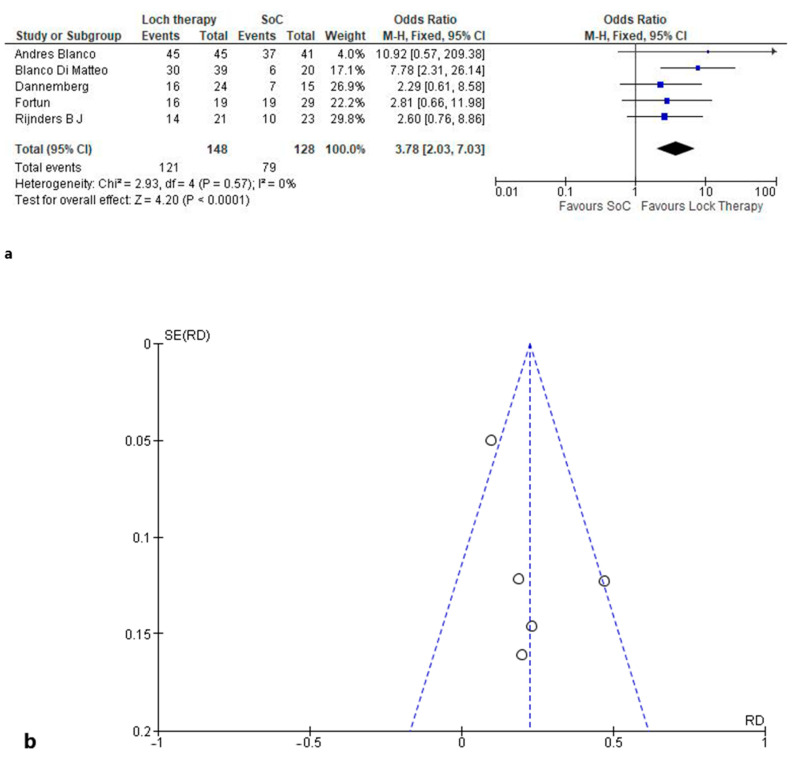
Effect of antibacterial lock therapy on end-of-treatment microbiological healing [8,9,10,11,12] (**a**) and funnel plot (**b**).

The end-of-treatment microbiological healing was analyzed in all five studies. A pooled analysis involving 276 participants showed an OR of 3.78 (95% CI; 2.03/7.03). The efficacy was evaluated in different follow-ups (from 2 weeks to 3 months). No heterogeneity was identified in the analysis (Chi^2^ = 2.93; *p* = 0.57; and I^2^ = 0%) (Figure 2). A funnel plot shows that only one study did not conform, which was easily explainable due to it being the only study that detected significant differences between the two groups [9]. The quality of evidence was very low, due to only observational studies being included.

#### 3.3.2. Adverse Events Favors

The adverse events were heterogeneously reported, and only the descriptive summaries can be assessed.

Mortality was only reported in the study by Fortun J [11], with only three deaths (1/19 vs. 2/19) being related to CVC infections.

CVC replacement was reported in three studies (Blanco-Di Matteo 2022 [9], Fortun J 2006 [11], Rijnders B.J. 2004 [12]), with a higher prevalence in the SoC groups in each study (Table 1). A mild liver enzyme increase was reported by Dannemberg C et al. [10].

## 4. Discussion

Our analysis showed significant differences between the efficacy of ALT and the SoC for microbiological healing, but no differences for adverse events were retrieved. This can improve the level of evidence on the use of ALT, which nowadays is limited to expert opinion [2]. We ought to highlight that all the retrieved studies were designed as observational studies, and our meta-analysis can only give very low-grade evidence results. The low heterogeneity across the studies can increase the likelihood level.

Looking at the evidence-based practice of ALT on CVC-related infections, the current guideline recommendations of 10–14 days of ALT are based on limited comparative clinical data. Furthermore, this practice has demonstrated significant benefits in hemodialysis patients and those with permanent CVCs for intravenous chemotherapy and TPN [13,14,15].

The efficacy of ALT has been mostly evaluated for prophylaxis in patients undergoing hemodialysis, parental nutrition, or antiblastic treatment [16,17,18]. Dialysis patients, due to the risk of clinical complications from CVCs, are more likely to manifest sepsis and CLABSIs [19]; thus, microbiological healing is important to allow the normal treatment of these patients, when replacement is not possible or suggested.

Furthermore, the prevention of CVC-related infections using ALT has been well evaluated in various population targets, from pediatric and oncological groups to elders [20], but few studies have evaluated microbiological healing in infected CVCs.

Only Blanco-Di Matteo et al. [9] showed significant differences in their study, as shown in Figure 2a, whereas the other studies showed only a trend. However, summing up their sample size and their impact on the meta-analysis, the significant difference on the efficacy of ALT was shown.

The profound impact of the complications associated with CVC use and how these complications can compromise the life expectancy of patients affected by chronic kidney disease on hemodialysis are well known. Even if the arteriovenous fistula on native vessels (AVF) is the first choice as the vascular access for hemodialysis, many patients do not have suitable vessels and the anatomical predisposition to be eligible for an AVF. In some patients, central vein catheters are the only option to ensure kidney replacement therapy when all the vascular resources have been compromised [21]. In these patients, it is essential to provide flawless management of the CVC to reduce the risk of infections and catheter malfunction.

Despite rare cases, CVC replacement is not possible due to the lack of any other usable vascular access. Furthermore, saving that specific access would reduce the amount of radio-interventional procedures in fragile patients, as well as hospitalizations. Indeed, in patients with a several risk of superior vena cava stenosis or inadequate flow, CVC replacement could be avoided [22]. Furthermore, critically ill patients are mostly subjected to femoral catheters, which are highly related to CVC infection, and CVC replacement should be avoided when possible [23].

Similarly, most of the observational studies evaluated the efficacy of ALT for microbiological healing, but they did not have a comparison. Thus, they could not give high-quality evidence despite the results showing a high success rate, because of the lack of a comparison [24,25,26,27,28,29]. All of these studies showed the highest occurrence of CVC salvage, but the evidence level of the uncontrolled observational studies did not give a clinical indication.

Although the efficacy of ALT compared to the SoC for the prevention of CVC-related infections [30,31] or to prevent reinfection in a replacement CVC [32] has been well studied, it is not enough to regulate ALT use when CVC-related infections are present.

The major limit of this meta-analysis is represented by the very low quality of the evidence, because no RCTs were published in the literature. The only RCT was focused on the prevention of infections [33].

Indeed, as shown in the specific guidelines about the prevention of catheter-related infections in subsection 11 of the 2019 KDOQI guidelines [2], a specific antiseptic solution was supported by very low-grade evidence (Table 2).

The very low quality of evidence is due to the lack of RCTs, despite the low heterogeneity of our meta-analysis. The grade of evidence derives mainly from the included study design, heterogeneity, and sample size. In keeping with this, RCTs are needed to improve the level of evidence and to suggest strong guidelines for the treatment of CVC-related infections [34,35].

This meta-analysis included patients that were afferent by various wards, such as hematology, oncology, gastroenterology, and nephrology. The presence of different kinds of patients gave our systematic review a high external relevance, due to the results being applicable and generalizable in various contexts.

Indeed, more RCTs on the treatment of CVC-related infections should be conducted, similar to the RCT on CVC-related infection prevention [17,33].

All studies, both those that were included and excluded, report high antimicrobial concentrations in ALT. This occurred because bacteria create an irreversible adhesion to a CVC surface in the process of the biofilm formation. This biofilm eradication requires a high antimicrobial concentration, as well as a long period before microbiological healing is reached [36].

Due to the small number of included studies, we could not separate based on population characteristics or treatment duration and could not perform other meta-analyses on these subgroups. As a result, no quantitative sensitivity analysis was performed.

## 5. Conclusions

Summing up, our results showed that ALT could improve the preservation of CVCs compared to the standard of care and could be considered when their replacement is not possible for vascular anomalies or to avoid invasive intervention in critical patients. Furthermore, adverse events such as death and CVC replacement were highly apparent in the standard of care groups, despite the reports not allowing a quantitative summary to be performed. Only observational studies were included, impairing the level of evidence in this systematic review. As a result, RCTs are needed to confirm these findings and to increase the level of evidence.

## Figures and Tables

**Table 1:** Summary of the main characteristics and findings of the RCTs reviewed.

Study, Year (Ref)	Study Population	Population Characteristics	Antibacterial Lock Therapy	Standard of Care	Study Duration	Outcome(s)	Results	Notes
Andres Blanco 2022 [8]	Patients with CVC-related infections Hemodialysis patients	N = 86 Age = 68 years Men = 57%	Teicoplanin 10 ng/mL Daptomycin 5 ng/mL Vancomycin 10 ng/mL N = 45	Heparin 500 IU N = 41	30 days	Microbiological healing	45/45 vs. 37/41	No adverse events were reported.
Blanco Di Matteo 2022 [9]	Pediatric patients with CVC-related infections Hemodialysis patients	N = 59 Age = 70 years Male = 33%	Teicoplanin 10 ngml Daptomycin 5 ng/mL Vancomycin 10 ng/mL N = 39	Heparin 500 IU N = 20		Microbiological healing	30/39 vs. 6/20	CVC replacement was needed in 4/39 vs. 9/20 patients. No other adverse events were reported.
Dannemberg 2003 [10]	Patients with CVC-related infections Oncological children and adolescents	N = 39 Age = 12 years Men = 57%	Ethanol N=24	Heparin N=15	28 days	Microbiological healing	16/24 vs. 7/15	Mild grade I elevation in the liver enzymes was noted in 8/24 vs. 3/15 patients.
Fortun J 2006 [11]	Patients with CVC-related infections Hospitalized patients with afferents by different wards.	N = 48 Age = 56 years Men = 40%	Vancomycin 2 ng/mL Gentamycin 2 ng/mL Ciprofloxacin 2 ng/mL N = 19	Heparin 20 IU/mL N = 29	14 days	Microbiological healing	16/19 vs. 19/29	CVC replacement was needed in 1/19 vs. 7/29 patients. Mortality was reported in 3/19 and 7/29. Only 1/19 and 2/29 deaths were related to CVC infections.
Rijnders B.J. 2004 [12]	Patients with CVC-related infections Hospitalized patients with afferents by different wards.	N = 44 Age = 48 years Men = not reported	Ceftazidime 500 mg/L Vancomycin 500 mg/L N = 21	Heparin 100 IU/mL N = 23	180 days	Microbiological healing	14/21 vs. 10/23	CVC replacement was needed in 3/21 vs. 9/23 patients. No other adverse events were reported.

**Table 2:** Summary of “KDOQI Clinical Practice Guideline for Vascular Access: 2019 Update. Guideline 11 Vascular Access Use” [2].

	Recommendation	Grade of Evidence
1	KDOQI considers it reasonable to assess or check the vascular access and surrounding area by physical exam prior to every cannulation (if AV access) or connection (if CVC) for potential complications.	Expert Opinion
2	KDOQI recommends rope ladder cannulation as the preferred cannulation technique for AVFs.	Conditional Recommendation, Moderate Quality of Evidence
3	KDOQI considers it reasonable to limit AV access through buttonhole cannulation only in special circumstances given the associated increased risks of infection and related adverse consequences.	Expert Opinion
4	KDOQI considers it reasonable to avoid buttonhole cannulation in synthetic PTFE grafts due to potential serious consequences.	Expert Opinion
5	KDOQI suggests that when select buttonhole cannulation is performed, the use of buttonhole cannulation devices to facilitate cannulation should be at the discretion and expertise of the cannulator.	Conditional Recommendation, Low Quality of Evidence
6	KDOQI considers it reasonable to use skilled cannulators with established high rates of cannulation success to perform the initial AV access cannulation on patients to help avoid primary infiltration injury of the AV access.	Expert Opinion
7	KDOQI considers it reasonable to have structured training and supervision of dialysis technicians and nurses before and during their initial cannulation attempts and regular training updates to maintain cannulation competency.	Expert Opinion
8	KDOQI considers it reasonable to support and educate eligible patients on self-cannulation of their AV access (AVF or AVG).	Expert Opinion
9	KDOQI suggests the use of a catheter care protocol for the exit site and hub care to reduce catheter-related bloodstream infections and the treatment of catheter dysfunction.	Strong Recommendation, Moderate Quality of Evidence
10	KDOQI considers it reasonable, in addition to correct hand hygiene/washing, to use aseptic techniques and masks for patients and staff performing the catheter connection and disconnection procedures.	Expert Opinion
11	KDOQI considers it reasonable to cleanse the catheter hub when connecting and disconnecting the catheter with a chlorhexidine-based solution. If chlorhexidine is contraindicated (e.g., sensitivity or allergy), a povidone–iodine solution (preferably with alcohol) is a reasonable substitute and should be used.	Expert Opinion
12	KDOQI considers it reasonable at the time of the change of the catheter dressing to cleanse the skin surrounding the catheter exit site with a chlorhexidine-based solution. If chlorhexidine is contraindicated (e.g., sensitivity or allergy), a povidone–iodine solution (preferably with alcohol) is a reasonable substitute and should be used.	Expert Opinion
13	There is inadequate evidence for KDOQI to make a recommendation on the specific chlorhexidine formulation to use for infection prophylaxis, and this should be based on the clinician’s best clinical judgment and local practical considerations.	NA
14	There is inadequate evidence to demonstrate a difference in catheter-related infections with the use of a transparent film dressing compared with a nontransparent dressing; thus, the choice of catheter dressing material should be based on the clinician’s discretion that considers the patient’s circumstances and uses best clinical judgment.	NA
15	KDOQI considers it reasonable to use a topical antiseptic or antibiotic barrier at the catheter exit site in addition to cleansing until the exit site is healed to reduce the risk of a catheter-related infection.	Expert Opinion
16	There is inadequate evidence to demonstrate a difference in catheter-related infections between the use of various antiseptic or antibiotic topical exit site barriers; thus, the choice of topical exit site barrier should be based on the clinician’s discretion and best clinical judgment.	NA
17	KDOQI considers it reasonable to follow these catheter care practices. The frequency of the change of the catheter dressing should be based on the clinician’s discretion and best clinical judgment, with a minimum of once-weekly catheter dressings that should be protected against wet and dirty environments, particularly when the exit site is not yet fully healed (e.g., swimming and showering should be avoided).	Expert Opinion

## Data Availability

The data that support the findings of this study are available from the corresponding author upon reasonable request.

## Supplementary Materials

The following supporting information can be downloaded at https://www.mdpi.com/article/10.3390/clinpract14040124/s1. Table S1: search strategy for PubMed and Embase.
