# Supplementary material for: Differences in Efficacy between Antibacterial Lock Therapy and the Standard of Care for CVC-Related Infections: A Systematic Review and Meta-Analysis
Source: Clin Pract. 2024 Aug 9;14(4):1538–49. doi: 10.3390/clinpract14040124 (PMC11352342; doi:10.3390/clinpract14040124)
Supplement: Supplementary file 1 [file clinpract-14-00124-s001.zip › clinpract-3109731-supplementary.pdf]

**Supplementary Table S1**. Search strategy PubMed and EMBASE
**PubMed (n=267)**
(((((catheter lock) OR (central venous catheter[MeSH Terms])) OR (central intravenous catheter[MeSH Terms]))) AND (infectious)) AND (OR (antibiotic*) OR (anti bacterial agent) OR (placebo))
**EMBASE (n=326)**
('central venous catheter'/exp OR 'catheter lock solution'/exp) AND 'infection'/exp AND 'daptomycin'/exp AND ('antiinfective agent'/exp OR 'placebo'/exp)
